# Intra- abdominal sepsis from a perforated duodenal ulcer—Management of a difficult surgical abdomen

**DOI:** 10.1016/j.ijscr.2019.01.033

**Published:** 2019-02-01

**Authors:** Mutua Irene, Mariga Denver, Marilyn Omondi, Kiptoon Dan

**Affiliations:** a Kampala International University, Uganda; b Department of Surgery, College of Health Sciences, University of Nairobi, Kenya

**Keywords:** Intra-abdominal sepsis, Peptic ulcer disease, Peritonitis, Open abdomen, Multiple abdominal surgeries

## Abstract

**Introduction:**

Peptic ulcer perforation is a common surgical emergency that is a complication of 9% of patients with peptic ulcer disease (Kjetil et al., 2015 [1]). Despite advances in the management of peptic ulcer disease, the number of acute complications have remained constant. Intra-abdominal sepsis is defined as inflammation of the peritoneum caused by pathogenic micro-organisms and their products (Pavlidis, 2003).

**Case presentation:**

We present a case report on intra-abdominal sepsis in a 20 yr old patient admitted in the intensive care unit at our institution following a perforated duodenal ulcer that was repaired by modified Graham patch method but leaked and he had multiple surgeries thereafter necessitating the open abdomen.

**Discussion:**

The open abdomen remains an option to the surgeon as the patient may have severe peritonitis or be in septic shock. Hence the need of damage control laparotomy due to severe physiological derangement or a planned second look laparotomy if there is failed source control.

**Conclusion:**

Intra-abdominal sepsis management of patient in an intensive care unit set-up with adequate surgery, open abdomen treatment and antibiotics given based on blood culture and sensitivity results enables successful management of difficult surgical abdomens.

## Highlights

• Management of intra- abdominal sepsis requires a multi-disciplinary approach for success to be achieved.
• Patient will undergo multiple surgeries and an open abdomen approach allows for planned re- laparotomies.

## 1 Introduction

The first summarized clinical description of the perforated peptic ulcer (50 cases) was made by Edward Crisp in 1843 [2]. Management of PUD has evolved over the decades with medical advances in operative techniques, microbiology and pharmacology. This has led to a decrease in elective surgery for PUD but has had no impact on the number of acute complications e.g ulcer perforations occurring in 9% of the patients [1] and bleeding requiring emergency surgery which have remained quantitatively constant [[3], [4], [5]]. The open abdomen (OA) procedure is one of the greatest surgical advances in recent times and is a useful option for treating patients with abdominal sepsis. Peritonitis occurs from loss of integrity of the gastrointestinal tract due to perforation or by direct invasion from infected intra-abdominal viscera. Peritonitis can lead to an excessive immune response and sepsis can quickly evolve into septic shock and multi organ failure [6,7]. Three strategies have been employed in the management of these difficult patients [8,9]:

• Re-laparotomy on demand (when required by the patient’s clinical condition).
• Planned re-laparotomy in the 36-48-h post-operative period (when re-laparotomy is planned after first operation).
• Open abdomen procedure.

These patients benefit from aggressive surgical treatment following an initial emergency laparotomy to control the local inflammatory response.

This case has been reported in line with the SCARE criteria [10].

## 2 Case presentation

A 20 yr old male patient was referred from a peripheral hospital to our institution intensive care unit having been operated on for perforated duodenal ulcer. He had presented with a long- standing history of peptic ulcer disease and had developed sudden onset of severe abdominal pain a day prior to admission. A plain radiograph done revealed free air under the diaphragm. Surgical intervention of a modified Grahams patch for a perforated duodenal ulcer was done. Post operatively, he was referred to due acute kidney injury(AKI).

At the ICU, his vital signs were an elevated heart rate of 140bpm, increased respiratory rate of 36 breaths per minute, low blood pressure of 80/50 mmHg and his temperature was 36.5°c. On examination, he had purulent discharge from his abdominal drain. His serum creatinine was elevated at 254 mmol/L (65–130) with an increased Blood Urea Nitrogen of 18.8 mmol/L (1.7–8.3). The arterial blood gas analysis (BGA): PH-7.354, PCO_2_ -3.57 kPa, PO_2_ – 8.26 kPa, HCO_3_ – 14.6 mmol/L, Base Excess- -9.6 mmol/l and SPO_2_ -91.3%. He was admitted with a diagnosis of hypovolaemic shock with intra-abdominal sepsis, compensated metabolic acidosis, and AKI. His initial management was intravenous fluids, empirical antibiotics: ceftriaxone, metronidazole and analgesics.

The patient was taken to theatre and intra operatively, 2 litres of purulent bilous material was found in the peritoneum and the previous repair had given way and there was now a large duodenal perforation with friable edges measuring approximately 1 cm in size at D1. A pyloric exclusion was done with a Roux-and-Y gastrojejunostomy and peritoneal lavage was done. Abdominal drains were left in-situ and he was taken back to the ICU.

The patient remained stable post operatively but was noted to have pus oozing from the incision site on post-op day three and on day four upon release of sutures he had a burst abdomen. He was also noted to be febrile with temperatures of 39 °C. An emergency laparotomy revealed a duodenal stump blow-out. Peritoneal lavage and repair of the duodenal stump was done and a “Bogota bag” as a method of temporary abdominal wall closure was chosen. He went to theatre twice weekly for peritoneal lavage and change of Bogota bag for an initial period of three weeks due to pus exudate from the abdomen and abdominal drains but as the effluent reduced, he went on demand basis. His clinical outlook was generally improving however the intra-abdominal infection persisted. The challenges we faced with the patient’s deranged physiology while in ICU are tabled as shown on Table 1.

**Table 1:** Laboratory values.

Dates	Neutrophil count	Haemoglobin	Creatinine	Urea
28/6/16	95.8%	10.3 g/dl	254 mmol/l	18.8 mmol/l
29/6/16	92%	10 g/dl	332 mmol/l	20 mmol/l
05/7/16	88%	12 g/dl	112 mmol/l	14 mmol/l

He was also noted to have a positive fluid balance on 28^th^ and 29^th^ and with a rising serum creatinine and a decision was made to start him on haemodialysis that saw his renal functions improve. He was transfused with blood as well.

Table 2 shows the microbial profile that was grown and the antibiotic sensitivity patterns. These are most likely hospital acquired infections. His urinary cultures only once grew candida and all his tracheal aspirates grew nothing on culture. His antibiotic treatment changed from the empirical treatment with ceftriaxone and metronidazole and was tailored as per microscopy, culture and sensitivity results. Throughout his stay in the ICU he was put on H.pylori treatment, thromboprophylaxis, total parenteral nutrition and two hourly turning in bed to avoid bed sores.

**Table 2:** Antibiotic therapy.

	Microbes grown	Antibiotic	Sensitive	Resistant
27/7/16	Candida spp	Fluconazole	S	
24/7/16	Citrobacter	Ciprofloxacin		R
		Cefotaxime		R
		Gentamicin		R
		Imipenem		R
		Cefuroxime		R
		Doxycycline		R
11/7/16	Acinetobacter baumanii	Levofloxacin	S	
		Cefuroxime		R
		Ceftriaxone		R
		Ciprofloxacin		R
		Augmentin		R
4/7/16	Pseudomonas aeruginosa	Ofloxacin		R
		Gentamicin		R
		Meropenem	S	

After the fifteen laparotomies of which 12 were planned, the repair held and the intra-abdominal sepsis was controlled. The patient developed an entero-atmospheric low output fistula that was managed conservatively. He started passing stool ten weeks after his admission and he was allowed to feed orally as the parenteral nutrition was weaned off. Granulation tissue formed over his bowel and the low output fistula eventually closed. The patient was transferred from ICU to the ward having stayed there for ten weeks. The wound continued to be dressed in the ward for the next 2 months and healed with formation of a large ventral wall hernia. He was discharged home to be reviewed by the plastic surgery team at the surgical outpatient clinic to plan for reconstruction of the abdominal wall at a later time.

## 3 Discussion

Intra-abdominal sepsis is defined as inflammation of the peritoneum caused by pathogenic micro-organisms and their products.^7^ Perforated duodenal ulcer results in secondary peritonitis as in our patient. Abdul et al in their study found out that intestinal perforation occurs more in males in a ratio of 3: 1 and perforated peptic ulcer accounted for 14% of the cases. Wound infection and intra-abdominal sepsis occurred in 57.4% and 8.5% respectively. Factors such as age, cause of intestinal perforation and amount of pus drained during an operation independently predicts the post- operative morbidity and mortality rates [11].

The patient also had acute kidney injury and it is theorized that gut mucosal hypoperfusion as an early consequence of hypovolaemia drives the intra-abdominal inflammatory process even when the initial causal factors are dealt with [6]. Thus, gut mucosal acidosis perpetuates leakage and may lead to intra-abdominal sepsis like in our patient.

Management of a patient with abdominal sepsis requires a multi-displinary team approach: surgeon, intensivist, microbiologist, pharmacologist, radiologist and a dedicated team of nurses preferably in an intensive care unit to maximize the chances of success in a physiologically prepared patient when operating on the underlying cause. Farthmann et al described three therapeutic principles in the surgical management of intra-abdominal infections: Elimination of the focus by controlling contamination source, contamination reduction to reduce or eliminate the bacterial load and finally treatment of residual and prevention of recurrent infection with antibiotics [9]. Source control importance supersedes the impact of antibiotic therapy [12].

Large bore abdominal drains are useful in intra- abdominal sepsis and should be placed in the appropriate dependent areas of the abdominal cavity like paracolic gutters, pelvis and sub-phrenic spaces away from the intestines [6]. Two abdominal drains were placed in our patient.

Closed and open lavage techniques, the open abdomen and the planned relaparotomy represent the major approaches in the management of intra- abdominal sepsis [9]. Open abdomen treatment in patients with peritonitis is increasing worldwide and offers added benefit in severely ill patients as part of damage control surgery [12,13]. Tolonen et al in their study found a median duration of open abdomen to be seven days with a median of two dressing changes [13] unlike in our case where the patient had an open abdomen for eight weeks and planned relaparotomies twelve times.

The most frequent indication for open abdomen as per the International Register of Open Abdomen (IROA) was peritonitis at 48.7% and the most adopted temporary abdominal closure technique was the commercial negative pressure wound therapy system at 44.2% then the Bogota bag and skin closure technique at 31.8% [14].Vacuum- assisted wound closure and mesh- mediated fascial traction (VAWCM) technique in patients with complicated secondary diffuse peritonitis and open abdomen yields excellent results in terms of delayed fascial closure rate and a low number of entero-atmospheric fistulas [13]. The longer the period of open abdomen, the more the complications including enteric fistulas with 10.5% of patients developing them as per IROA [14]. Our patient developed a low output entero-atmospheric fistula which spontaneously closed over time. This increased his intensive care unit – hospital stay and thus cost.

Bogota bag was placed in our patient as a means of temporary abdominal closure since it is readily available, cheap, non- allergic, non- adherent to the gut and easily sutured to the abdominal wall skin. It also allows easy approach to the abdomen during re- laparotomy.

The patient recovered from the intra-abdominal sepsis successfully but not without development of a large ventral hernia as the fascial tissues closed over time and this constitutes one of the difficult post- operative problems requiring future solutions by the plastic surgery team.

## 4 Conclusion

Management of patients who have developed intra-abdominal sepsis in an intensive care unit set-up, adequate surgery, open abdomen treatment and antibiotics given based on blood culture and sensitivity results enables successful management of difficult surgical abdomens.

## Conflicts of interest

All authors: Irene Mutua, Denver Mariga, Marilyn Omondi and Dan Kiptoon have no conflict of interest.

## Sources of funding

No funding or grant support.

## Ethical approval

This is a case report and it is exempted from ethical approval in the University of Nairobi.

## Consent

Written informed consent was obtained from the patient for publication of this case report. This report does not contain any personal information that could lead to the identification of the patient. A copy of the written consent is available for review by the Editor- in Chief of this journal on request.

## Author contribution

1 Dr. Irene Mutua- Pediatric Surgery resident, Department of Surgery, College of Health Sciences, P.O Box 19676-00202, University of Nairobi Kenya. Mobile Phone: 0711261324 Email: immutua@yahoo.com.

I am the corresponding author.

2 Dr Denver Mariga- Pursing Master of Science in Field Epidemiology, P.O Box 3900- 30100, Moi University, Eldoret, Kenya.

Was involved in drafting the manuscript and its critical revision.

3 Dr Marilyn Omondi- Tutorial Fellow and Consultant General Surgeon, Department of Surgery, College of Health Sciences, P.O Box 19676-00202, University of Nairobi, Kenya.

Was involved in the management of the patient, drafting the manuscript and its critical revision.

4 Dr Dan Kiptoon- Lecturer and Consultant General Surgeon, Department of Surgery, College of Health Sciences, P.O Box 19676-00202, University of Nairobi, Kenya.

Was involved in the case report conception, management of the patient, drafting the manuscript and its critical revision.

## Registration of research studies

This is a case report of a patient we managed and not a human study research.

## Guarantor

Dr Dan Kiptoon.

Email: dkiptoon@gmail.com.

## Provenance and peer review

Commissioned, externally peer-reviewed.
